# Investigation of the Ability to Detect Electrolyte Disorder Using PET with Positron Annihilation Lifetime Spectroscopy

**DOI:** 10.1021/acs.jpcb.3c04208

**Published:** 2023-11-10

**Authors:** Radosław Zaleski, Olga Kotowicz, Agnieszka Górska, Kamil Zaleski, Bożena Zgardzińska

**Affiliations:** † Maria Curie-Sklodowska University, Institute of Physics, Department of Material Physics, Pl. M. Curie-Sklodowskiej 1, 20-031 Lublin, Poland; ‡ Medical University of Lublin, Faculty of Medicine, Clinic of Toxicology, Al. Kraśnicka 100, 20-718 Lublin, Poland

## Abstract

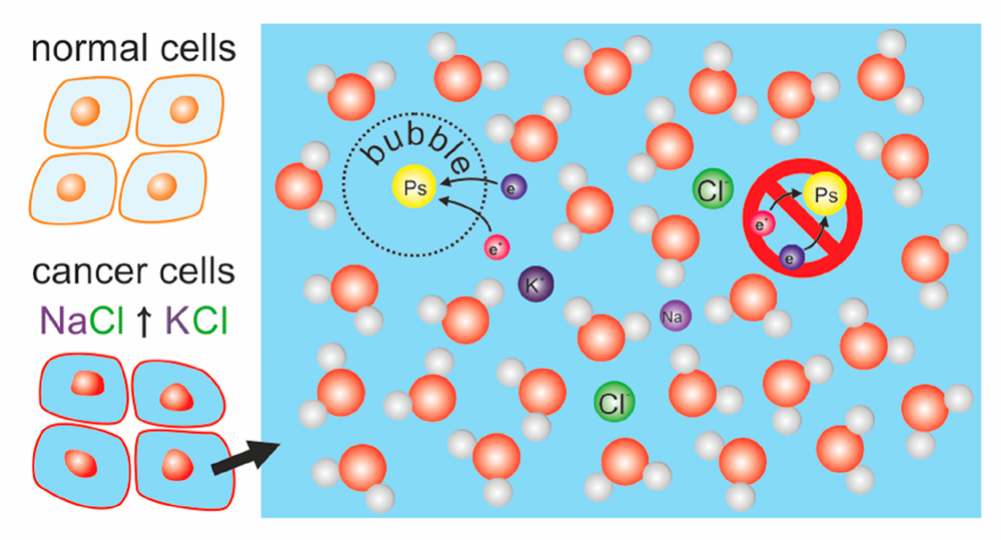

Various concentrations (8–300 mmol/L) of NaCl, KCl, and NaCl + KCl aqueous solutions were investigated using positron annihilation lifetime spectroscopy (PALS). A strong dependence of the *o*-Ps intensity as a function of the solution concentration was demonstrated. On this basis, the mean positron lifetime and the sum of counts in a selected time interval were proposed as reliable parameters for detecting disturbances in the ion balance of living organisms. The use of these parameters for differentiating healthy and cancerous tissues allows for the development of auxiliary diagnostic methods in a new generation of PET scanners equipped with a PALS detection module.

## Introduction

Hyponatremia and hypokalemia are the two most frequent electrolyte disorders encountered in cancer patients.^1,2^ It has also been established that the concentration of electrolytes is different in cancer tissue than in healthy tissue. Tumorous cells have significantly higher intracellular concentrations of sodium and potassium.^3^ According to the cellular excitability (“CELEX”) hypothesis, based on electrophysiological properties of cell membranes, strongly metastatic cancer cells have more electrically excitable membranes which makes them aggressive.^4^ Overexpression of VGSCs (voltage-gated sodium channels) in many types of cancer causes an increase in Na^+^ influx, which can be linked to mechanisms that increase their invasiveness and metastasis.^5^ VGSCs are up-regulated only in strongly metastatic cells. VGPCs (voltage-gated potassium channels) are downregulated in this phase but play a role in proliferation. Thus their activity increases in cells that have reached metastatic sites and grow into secondary tumors. The role of VGSCs is better recognized and can be targeted by anti-metastatic treatment.^6^ A higher concentration of sodium in tumor tissue was already measured by magnetic resonance imaging.^7,8^

A recently developed total body PET has the ability to measure positron annihilation lifetimes.^9−11^ This technology, in addition to the geometric reconstruction of positron annihilation sites, allows one to obtain information about the probability of formation and annihilation of positronium, the unstable bound state of an electron and positron. The annihilation parameters (lifetime and intensity) of the component in the positron lifetime spectrum, which corresponds to the triplet state of positronium (*ortho*-positronium, *o*-Ps), correlate with the structure of the medium (e.g., the size of the space between molecules can be determined) and its chemical environment, (e.g., it is expected to detect hypoxia^12^). The relationship between the degree of lesions, the type of the investigated tissue, and these parameters was discussed.^13−15^ However, the lack of measurements for model systems makes it difficult to understand the origin of changes in the annihilation parameters. The aim of this paper is to investigate whether fluctuations in electrolyte concentration are possible to detect with positron annihilation lifetime spectroscopy (PALS).

## Methods

A digital positron lifetime spectrometer was used for the PALS measurements. It was equipped with an Agilent U1065A digitizer with a sampling rate of 4 GS/s and the software^16^ dedicated to the analysis of pulses from two scintillation detectors equipped with BaF_2_ scintillators. The source of positrons was ^22^NaCl with an activity of 0.5 MBq placed in an 8 μm thick Kapton envelope immersed in the center of a cylindrical container with a diameter of 16 mm and made of PTFE. A liquid was kept at body temperature (309.6 K) after degassing with the freeze–pump–thaw technique. For each sample, the positron lifetime spectrum was collected with the number of counts around 1.3 × 10^7^. The spectra were analyzed using PALSfit software. A source correction with an intensity of 9.7% and a lifetime of 382 ps and a resolution function approximated by a single Gaussian with a FWHM of 189 ps were assumed. Three components originating from *para*-positronium (*p*-Ps), unbound positrons, and *ortho*-positronium (*o*-Ps) were assumed. This resulted in good fits with χ^2^ < 1.1.

Ultrahigh purity H_2_O (18 MΩ at 298 K) was used to prepare water solutions of NaCl and KCl (POOCH, Poland, p.a.) at concentrations of 8, 16, 25, 50, 75, 150, and 300 mmol/L and their 1:1 mixture at concentrations of 16 and 150 mmol/L.

## Results and Discussion

**Figure 1:**
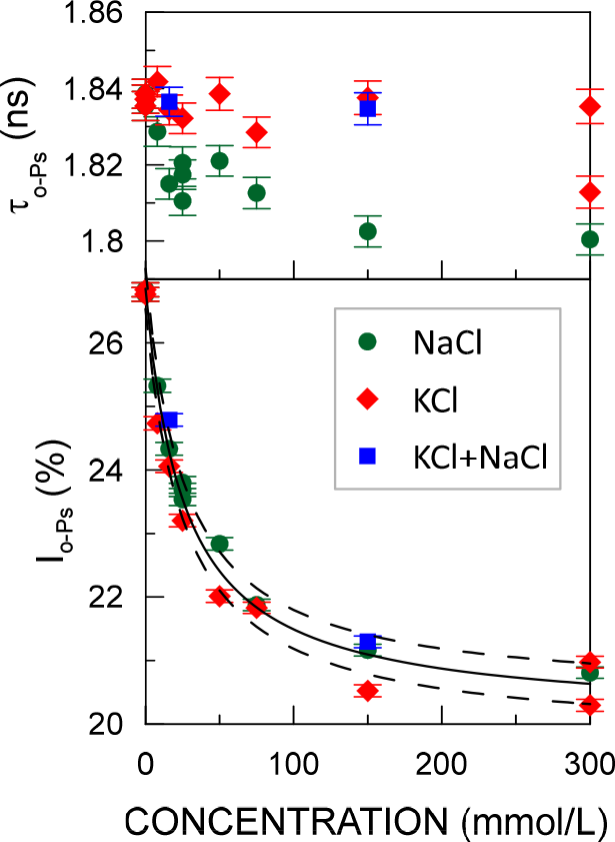
*ortho*-Positronium lifetimes and intensities as a function of the concentration of water solutions of NaCl, KCl, and their 1:1 mixture. The solid line represents the curve fitted with eq 1, and the dashed curves represent the confidence interval with a standard deviation.

No significant change in the electrolyte concentration was found in the *p*-Ps lifetime. Its mean value was 244 ± 6 ps, i.e., with a standard deviation of all results of 6 ps. This value is significantly different from the *p*-Ps lifetime in a vacuum of 125 ps. Most likely this is a result of radiolytic processes^17^ or the formation of the quasi free Ps,^18^ which also distorts the expected *p*-Ps to *o*-Ps ratio of 1:3. The lifetime of unbound positrons shows a small decrease from 500 ± 3 ps to its mean value above 16 mmol/L of 467 ± 8 ps. The mean lifetime of *o*-Ps (τ_*o*-Ps_) is 1.83 ± 1 ns, but possibly the lifetimes for NaCl solutions are slightly smaller (Figure 1). The most pronounced change is in intensities: the *o*-Ps intensity (*I*_*o*-Ps_) decreases from (27.0 ± 0.1)% to (20.3 ± 0.1)% (Figure 1). The *I*_*o*-Ps_ strongly depends on the electrolyte concentration and is weakly dependent on the type of water-electrolyte solutions.

This is accompanied by a decrease in *p*-Ps intensity from (35.2 ± 0.9)% to (25.1 ± 1.2)% and an increase in unbound positron intensity from (37.9 ± 1.1)% to (54.1 ± 0.8)%. The intensity change with the salt concentration is the same for NaCl and KCl as well as the 1:1 mixtures. This allows one to assume that the intensity decreases due to the inhibition of positronium formation by the chloride ions.^19^ This implies that PALS will hardly distinguish between sodium and potassium salts, but it is sensitive to the concentration of chlorides.

The intensity dependence on the chloride concentration is very well fitted (Figure 1) using the equation
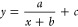
1where *y* is *I*_*o*-Ps_ in %, *x* is the electrolyte concentration in mmol/L, *a* = (176 ± 20)% × mmol/L, *b* = (26 ± 3) mmol/L, and *c* = (20.1 ± 0.2)% are the fitting parameters.

The intracellular chloride concentration usually does not exceed 60 mmol/L, which corresponds to *I*_*o*-Ps_ > 22% calculated using eq 1. The studies of cancerous tissues show that this concentration can increase even 2–3 times.^20,21^ This, in turn, corresponds to *I*_*o*-Ps_ < 21%. This difference is not large but exceeds the standard deviation of *I*_*o*-Ps_ by 10 times. Moreover, an imbalance in chloride concentration is expected to reduce chloride levels in the tissue surrounding the cancer to below normal levels. This would result in a distinct increase in *I*_*o*-Ps_ outside the cancer and, consequently, a marked step in intensity at the border of the cancerous tissue.

**Figure 2:**
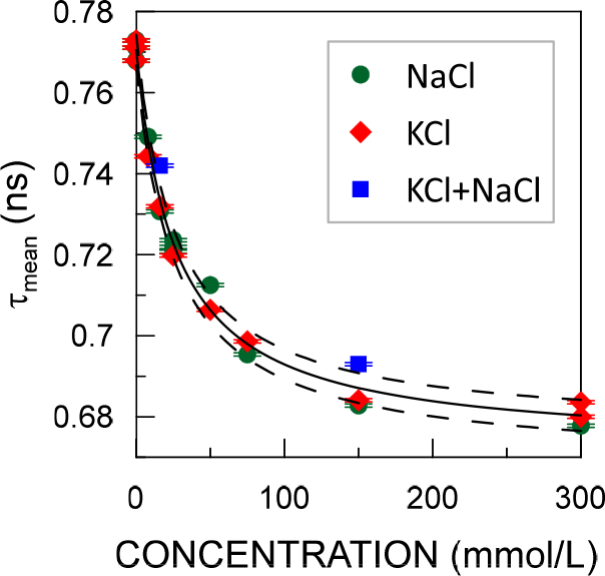
Mean positron lifetime as a function of the concentration of water solutions of KCl, NaCl, and their 1:1 mixture. The solid line represents the curve fitted with eq 1, and the dashed curves represent the confidence interval with a standard deviation.

The ratio of the difference between healthy and cancerous tissue to the experimental uncertainty can be even increased by using eq 1 to fit the dependence of the mean positron lifetime (τ_mean_) on the chloride concentration (Figure 2). τ_mean_ is given by the equation
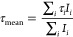
2where *i* are indices of all positron components in the spectrum. The mean positron lifetime is known to be a very reliable parameter that is almost independent of inaccuracies in the fitting (e.g., problems in resolving spectra into components if their lifetimes are too close). Using τ_mean_ as *y* in eq 1 results in the following fitting parameters: *a* = (2.6 ± 0.2 ns) × mmol/L, *b* = 27 ± 2, mmol/L, and *c* = 0.672 ± 0.002 ns. Normal intracellular chloride concentration is indicated by τ_mean_ > 0.699 ns, while in cancer τ_mean_ < 0.686 ns. This difference exceeds the standard deviation of τ_mean_ by over 30 times. The scatter of the experimental results exceeding three standard deviations is negligible. Furthermore, a full body scan with PET yields multiple spectra within the cancerous tissue as well as the surrounding healthy tissue. Each of them consists of a high number of counts due to high source activity (∼GBq). Therefore, there should be a clear statistical difference in τ_mean_ due to the variations in chloride concentrations.

**Figure 3:**
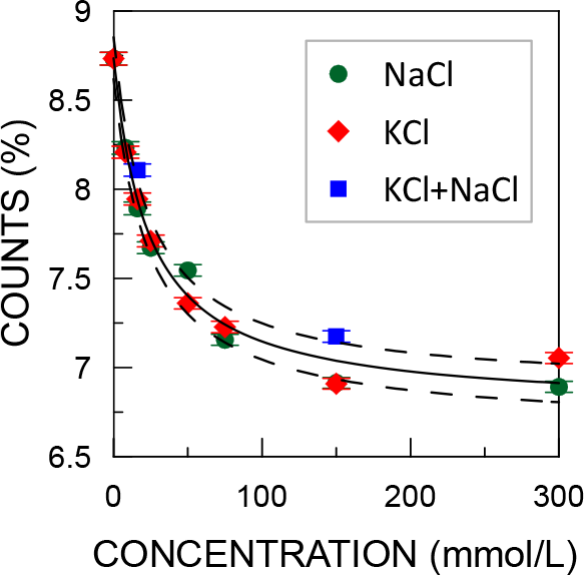
Sum of counts in the 2–12 ns time interval of the positron lifetime spectra as a function of the concentration of water solutions of KCl, NaCl, and their 1:1 mixture. The solid line represents the curve fitted with eq 1, and the dashed curves represent the confidence interval with a standard deviation.

Instead of the typical analysis of positron lifetime spectra, only the sum of counts in the 2–12 ns time interval of the spectrum (*N*_2–12ns_) can be taken into account. This part of the spectrum contains 7–9% of the total counts (Figure 3), which originate almost entirely from the *o*-Ps component. The contribution of other components above 2 ns is 50 times smaller than the *o*-Ps component; thus, their changes are negligible for *N*_2–12ns_. There is also a constant contribution from the background of random coincidences, but it does not exceed 0.5%.

This approach does not require any fitting procedure and therefore allows uncertainties comparable to the uncertainties of *I*_*o*-Ps_ to be obtained with a 16 times smaller number of total counts, i.e., 8 × 10^6^, in a spectrum. Such a number of total counts was collected during 1 h. This allowed the time stability of *I*_*o*-Ps_ to be studied. There were no systematic changes in *N*_2–12ns_ over time for concentrations up to 150 mmol. Only for the concentration of 300 mmol/L was a slow linear decrease of *N*_2–12ns_ with a slope of −0.005(2)%/h observed. This may be due to the accumulation of water radiolysis products over time, which react with Cl^–^ modifying its inhibition rate. This effect is insignificant for the measurement results.

Using the number of counts as *y* in eq 1 results in the following fitting parameters: *a* = 46 ± 7, *b* = 23 ± 3, and *c* = 6.77 ± 0.06.

## Conclusions

There is increasing evidence of substantial differences in the expression of ion channels and electrolyte concentrations between cancer tissue and healthy ones.^1−5^ This provides an opportunity to use these differences for both diagnostics and therapeutic purposes under development.^6^ The combination of PET imaging with positron annihilation lifetime spectroscopy^9−11^ introduces new possibilities for identifying cancerous tissue, e.g., based on the concentration of Cl^–^ ions. Differences in the Cl concentration between healthy tissue and cancer are large enough to be statistically significant in PALS measurements. In particular, the use of the mean positron lifetime or the sum of counts in a selected time interval of the spectrum allows small uncertainties to be obtained of the results. Note that these measurements should be from live cells, when the chloride transport mechanisms are active. Moreover, there are also other factors influencing the positron lifetime spectra, which together with electrolyte disturbances influence the positron lifetime spectra. However, each of these factors has a slightly different impact (e.g., hypoxia, which mainly affects lifetime). This allows us to expect that the comparison of experimental data with a model taking into account the most important factors will provide important diagnostic information.

Additionally, recent studies suggest synthetic anion transporters can induce apoptosis and can be used in cancer treatments.^22^ The presented method can be used in in vivo studies of such a treatment or monitoring its progress.
